# Main and contralateral side stages of lesion affected bone union in the conservative treatment of adolescent lumbar spondylolysis: a multivariable analysis of 217 patients and 298 lesions in a retrospective cohort study

**DOI:** 10.1186/s13018-023-03861-y

**Published:** 2023-06-03

**Authors:** Hisanori Gamada, Masaki Tatsumura, Reo Asai, Shun Okuwaki, Toru Funayama, Masashi Yamazaki

**Affiliations:** 1 Department of Orthopedic Surgery, Faculty of Medicine, University of Tsukuba, 1-1-1 Tennodai, Tsukuba, Ibaraki 305-8575 Japan; 2 Department of Orthopedic Surgery and Sports Medicine, Tsukuba University Hospital Mito Clinical Education and Training Center/Mito Kyodo General Hospital, 3-2-7 Miyamachi, Mito, Ibaraki 310-0015 Japan

**Keywords:** Adolescent, Bone union, Conservative treatment, Lumbar spondylolysis, Multivariate logistic regression analysis

## Abstract

**Background:**

Factors affecting bone union in the conservative treatment of adolescent lumbar spondylolysis remain controversial. We aimed to examine these factors along with advances in diagnostic imaging using multivariable analysis of a sufficient number of patients and lesions.

**Methods:**

In this retrospective study, high-school-aged patients or younger (*n* = 514) who were diagnosed with lumbar spondylolysis from 2014 to 2021 were investigated. We included patients with acute fractures who showed signal changes around the pedicle on magnetic resonance imaging and who completed conservative treatment. The following factors were investigated at the initial visit: age, sex, level of lesion, main side stage, presence and stage of contralateral side lesion, and presence of spina bifida occulta. The association of each factor with bone union was evaluated through a multivariable analysis.

**Results:**

Altogether, 298 lesions in 217 patients (174 boys and 43 girls; mean age: 14.3 years) were included in this study. Multivariable logistic regression analysis of all factors showed that the main side progressive stage was more likely associated with nonunion as compared to the pre-lysis (OR: 5.86; 95% confidence interval [CI]: 2.00–18.8; *p* = 0.0011) and early stages (OR: 3.77; 95% CI: 1.72–8.46; *p* = 0.0009). Regarding the contralateral side stage, the terminal stage was more likely to be associated with nonunion.

**Conclusion:**

In the conservative treatment of lumbar spondylolysis, the factors affecting bone union were the main and contralateral side stages. Sex, age, level of lesion, or spina bifida occulta had no significant effects on bone union. The main, progressive, and contralateral side terminal stages were negative predictors of bone union.

*Trial registration* This study was retrospectively registered.

**Supplementary Information:**

The online version contains supplementary material available at 10.1186/s13018-023-03861-y.

## Introduction

Lumbar spondylolysis is a common fatigue fracture of the lumbar pars interarticularis in young athletes [1, 2]. Conservative treatment is the gold-standard treatment for acute fractures, in which lesions showing high-signal changes around the pedicle are identified in short-tau inversion recovery magnetic resonance imaging (MRI). This results in successful bone union in 77–100% of patients [1, 3–5]. However, while this bone union rate is high, surgery may be required in cases where bone union is not achieved, leading to pseudoarthrosis [6–8]. With conservative treatment, the factor most likely to affect bone union is the lesion stage.

However, several recent reports reported the following factors that affect bone union: whether the lesion is unilateral or bilateral, the presence of contralateral pseudoarthrosis, and the patient’s age [3, 9–12]. Although few reports have comprehensively evaluated the association of these factors with bone union via a multivariable analysis, these studies were not reported most recently [12, 13]. It is questionable whether the findings of these past reports can be applied now that the diagnostic methods using MRI and computed tomography (CT) reconstruction images and methods for observing the course of conservative treatment have advanced [3, 9, 12–14].

Therefore, the factors that predict the patient's bone union rate at the initial visit are still controversial, except for the stage of the lesion. In this study, we included staging in accordance with recent diagnostic advances in CT and MRI and a sufficient number of patients and lesions as important novelties. This study aimed to examine the factors affecting bone union in conservative treatment along with advances in diagnostic imaging through a multivariable analysis of a sufficient number of patients and lesions.

## Methods

We retrospectively investigated high-school-aged patients or younger (*n* = 514) diagnosed with lumbar spondylolysis between April 2014 and March 2021 at a single center. All the patients were symptomatic and presented with lower back pain. Asymptomatic patients diagnosed incidentally on imaging studies were not included in the sample.

We included acute fracture patients whose short-tau inversion recovery MRI scans showed high-signal changes around the pedicle [14] and who had completed conservative treatment according to the protocol described elsewhere [15]. High MRI signal changes on both pedicles were counted as two lesions. The exclusion criteria were as follows: patients with terminal stage lesions alone at the initial visit who were not eligible for conservative treatment targeting bone union, those who changed hospitals during treatment, those who dropped out of the treatment protocol (e.g., refused to wear a brace or returned to exercise at their own discretion during conservative treatment), and patients with missing data.

Plain radiography and MRI were performed for all patients at baseline. If high-signal changes were found around the pedicle and the patient was diagnosed with lumbar spondylolysis, CT was performed to assess the stage of the lesions. The protocol for conservative treatment is exercise prohibition, including physical education, wearing a semi-rigid extension-blocking brace, and undergoing athletic rehabilitation [15]. During conservative treatment, MRI was performed once a month to evaluate high-signal changes around the pedicle, and CT was performed to evaluate bone union when the high-signal changes on MRI disappeared. Patients were allowed to resume exercise gradually when the signal changes on MRI disappeared [3, 15]. The same conservative treatment protocols, intervals, and sequence of imaging studies were used for all patients. Patients with missing imaging studies data or those who did not follow the protocol were excluded from the study as described above. The treatment period was defined as the period from the date of diagnosis to the date when the high-signal changes disappeared on MRI. Bone union in this study was defined as the continuity of cortical bone in at least two different planes on multiplanar reconstructed CT images [9–11, 15]. The MRI and CT evaluations for this study were performed by two spine surgeons who assessed the images separately and discussed any disagreements. Information on patients’ daily participation in sports activities was collected.

**Fig. 1:**
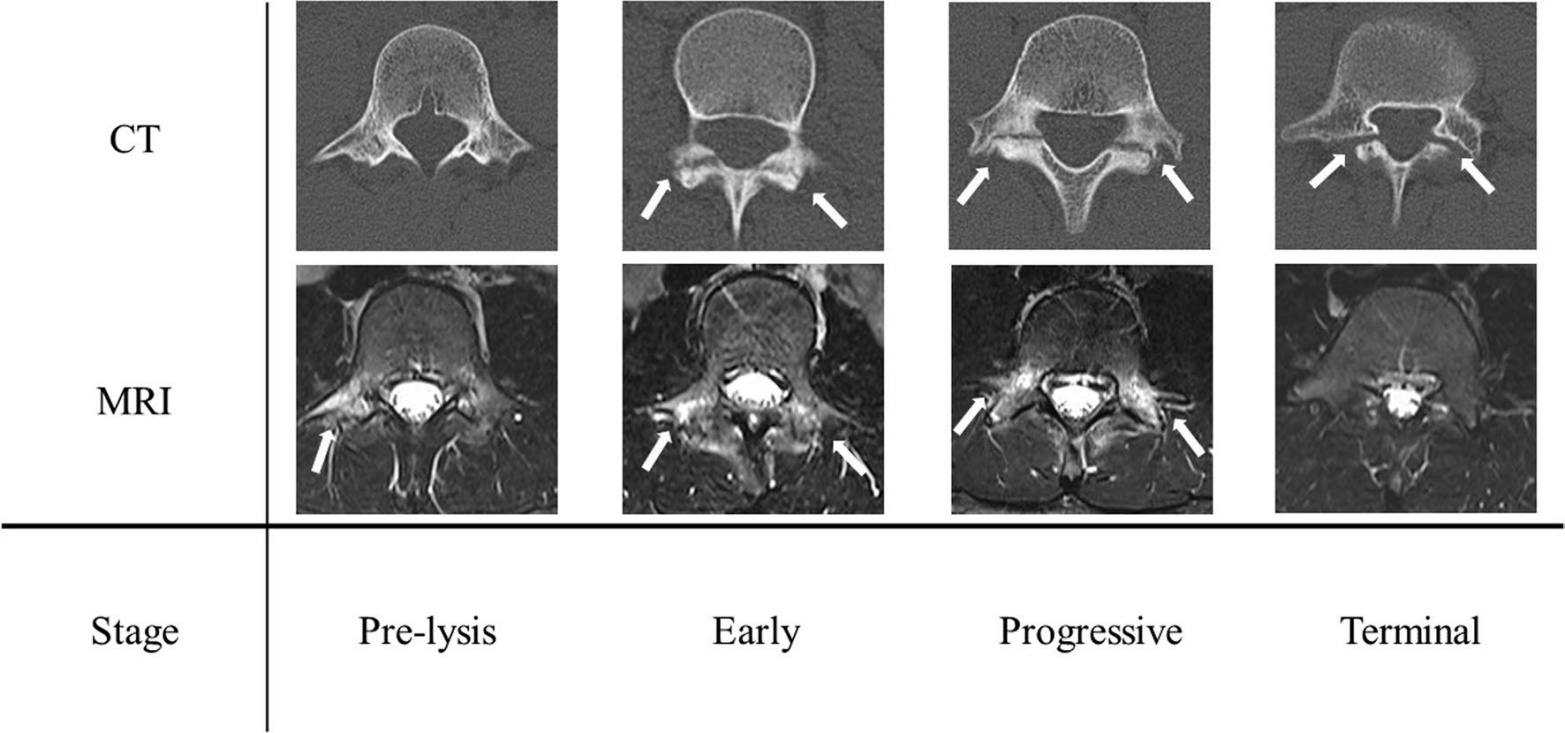
Explanation of stage via MRI and CT. Pre-lysis stage: high-signal change on MRI (arrow) without a fracture line on CT. Early stage: partial gap (arrow) on CT with high-signal change on MRI (arrow). Progressive stage: clear gap on CT (arrow) with a high-signal change on MRI (arrow). Terminal stage: a gap on CT (arrow) without a signal change on MRI, which was considered pseudoarthrosis. CT, computed tomography; MRI, magnetic resonance imaging

According to previous reports, the following factors that might influence the treatment results were investigated at the time of initial visit: sex, age, level of lesion, main side stage on the CT axial view (pre-lysis stage, Hollenberg grade 1: high-signal change on MRI without a fracture line on CT; early stage, Hollenberg grade 2: partial gap on CT with high-signal change on MRI; progressive stage, Hollenberg grade 3: clear gap on CT with a high-signal change on MRI; and terminal stage, Hollenberg grade 4: a gap on CT without a signal change on MRI, which was considered pseudoarthrosis) (Fig. 1), presence and stage of the contralateral side lesion, and presence of spina bifida occulta (SBO) in the lumbosacral spine that is not limited to the lamina with lesions [3, 9–13, 16, 17]. If the lesion was unilateral, the contralateral stage was evaluated as “none.” If the lesion was bilateral, it was evaluated as two lesions, and the main and contralateral stages were evaluated for each lesion. The association of each factor with bone union after conservative treatment was evaluated.

### Statistical analysis

Multivariable logistic regression analysis was performed using six factors at the time of the initial visit (sex, age, level of lesion, main side stage, contralateral side stage, and presence of SBO) as the explanatory variables and the presence of bone union as the objective variable. Age (< 13 years and ≥ 13 years) and the level of lesion (L5 or non-L5 [L2–L4]) were used as dichotomous variables, whereas sex, main side stage, contralateral side stage, and presence of SBO were used as nominal variables based on previous reports [10–12]. The number of explanatory variables was set to ensure that the number of lesions that had not achieved bone union was greater than the number of explanatory variables × 10. Odds ratios (ORs) of nonunion after conservative treatment were calculated. For the significant factors extracted from the multivariable analysis, we performed another multivariable analysis by dividing the factors into the groups of positive, intermediate, and negative factors for bone union. The significance level was set at *p* < 0.05. Statistical analyses were performed using JMP^®^ 10 (SAS Inc., Cary, NC).

## Results

**Fig. 2:**
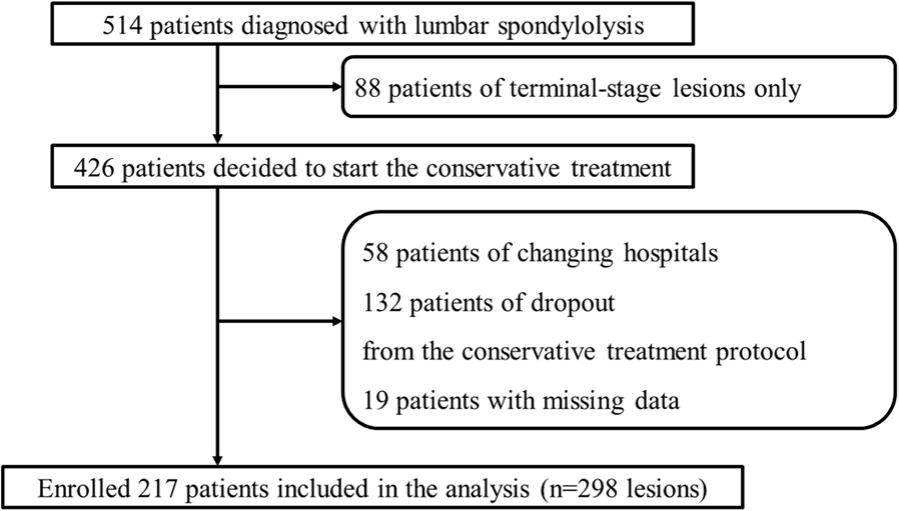
Inclusion and exclusion criteria

Overall, 298 lesions of 217 patients (174 boys and 43 girls; mean age: 14.3 years) were included in this study (Fig. 2). The conservative treatment protocol dropout rate was 26% (132 patients). This included 54 failures to maintain hospital visits and 78 protocol deviations, such as returning to sports or removing their brace before the resolution of MRI signal changes.

Among our sample, 214 patients (99%) participated in sports activities on a daily basis. By type of sport, soccer was the most common, with 58 patients (27%), followed by baseball, with 55 patients (25%). Three patients (1.4%) didn’t play any sports and participated in organized exercise only during school physical education (Table 1).

**Table 1:** Sporting activities of the patients in this study

Type of sport	*N*
Participants	214
Soccer	58
Baseball	55
Track and field	22
Volleyball	19
Basketball	13
Tennis	12
Softball	8
Swimming	8
Other	22
Physical education at school only	3

The total number includes three patients who played two sports

### Overall characteristics and proportion of lesions achieving bone union

The overall characteristics and proportion of lesions achieving bone union are shown in Table 2. The proportion of lesions achieving bone union was 77%, and the overall mean treatment period was 107 days. Except for SBO, no patients had an obvious anomaly.

**Table 2:** Overall characteristics and proportion of lesions that achieved bone union

	Total	Bone union	Proportion of lesions that achieved bone union (%)
All lesions	298	229	77
*Sex*			
Male	242	183	76
Female	56	46	82
*Age*			
< 13 years	55	37	67
≥ 13 years	243	192	79
*Level of lesion*			
Non-L5 (L2–L4)	107	90	84
L5	191	139	73
*Main side stage*			
Pre-lysis	92	84	91
Early	136	115	85
Progressive	70	30	43
*Contralateral side stage*			
None	113	110	97
Pre-lysis	35	26	74
Early	66	55	83
Progressive	47	28	60
Terminal	37	10	27
*Spina bifida occulta*			
Without	144	117	81
With	154	112	73

### Multivariable logistic regression analysis

The results of the multivariable logistic regression analysis of sex, age, level of lesion, main side stage, contralateral side stage, and presence of SBO are shown in Table 3. No significant differences were observed in sex, age, level of the lesion, or the presence or absence of SBO. The main side progressive stage was more likely to be associated with nonunion than to pre-lysis (OR: 5.86; 95% confidence interval (CI): 2.00–18.8; *p* = 0.0011) and early stages (OR: 3.77; 95% CI: 1.72–8.46; *p* = 0.0009) (Additional file 1).

**Table 3:** Multivariable logistic regression analysis of each factor

	Reference	OR	95% CI	*p*
*Sex*				
Female	Male	0.6	0.22–1.55	0.30
Age				
≥ 13 years	< 13 years	0.73	0.31–1.72	0.47
Level of lesion				
L5	Non-L5 (L2–L4)	1.15	0.53–2.54	0.72
Main side stage				
Early	Pre-lysis	1.55	0.57–4.52	0.39
Progressive	Pre-lysis	5.86	2.00–18.8	**0.0011**
	Early	3.77	1.72–8.46	**0.0009**
Contralateral side stage				
Pre-lysis	None	12.8	3.39–63.1	**0.0001**
Early	None	5.17	1.43–24.6	**0.011**
	Pre-lysis	0.40	0.13–1.25	0.11
Progressive	None	9.74	2.67–47.1	**0.0004**
	Pre-lysis	0.76	0.24–2.44	0.64
	Early	1.88	0.72–4.97	0.19
Terminal	None	52.7	14.2–264	**< 0.0001**
	Pre-lysis	4.12	1.27–14.2	**0.018**
	Early	10.2	3.66–30.9	**< 0.0001**
	Progressive	5.42	1.99–15.9	**0.0008**
Spina bifida occulta				
With	Without	1.50	0.74–3.08	0.26

*OR* odds ratio; *CI* confidence interval

Bold indicates statistically significant at *p* < 0.05

Regarding the contralateral side stage, the terminal stage was more likely to be associated with nonunion, whereas the absence of a contralateral side lesion (unilateral lesion) was more likely to be associated with bone union. The contralateral side’s pre-lysis, early, and progressive stages were intermediate.

### Multivariable logistic regression analysis in the main and contralateral side stages

Based on the results of the multivariable logistic regression analysis, it was considered that the progressive stage in the main side and the terminal stage in the contralateral side were negative factors for bone union; the pre-lysis, early, and progressive stages in the contralateral side were intermediate factors; and the pre-lysis and early stages in the main side and absence of contralateral side lesion (unilateral lesion) were positive factors for bone union. Multivariable logistic regression analysis was performed by dividing the lesions into groups stratified by main (progressive vs. pre-lysis and early) and contralateral (terminal vs. pre-lysis, early, and progressive vs. none) side stages (Table 4). Significant differences were found in all parameters (*p* < 0.0001).

**Table 4:** Multivariable logistic regression analysis of main and contralateral side stages

	As reference	OR	95% CI	*p*
Main side stage				
Progressive	Pre-lysis and early	4.56	2.31–9.15	**< 0.0001**
Contralateral side stage				
Pre-lysis, early, and progressive	None	8.30	2.78–35.8	**< 0.0001**
Terminal	None	53.1	14.8–260	**< 0.0001**
	Pre-lysis, early, and progressive	6.40	2.78–15.7	**< 0.0001**

*OR* odds ratio; *CI* confidence interval

Bold indicates statistically significant at *p* < 0.05

## Discussion

In this study, multivariable logistic regression analysis was performed to investigate the factors affecting bone union of acute fractures in adolescent patients receiving conservative treatment for lumbar spondylolysis. We found that the factors affecting bone union were the main and contralateral side stages. Sex, age, level of lesion, and SBO had no effect on bone union. Notably, the main side progressive and contralateral side terminal stages were the negative factors for achieving bone union. In contrast, main side pre-lysis and early stages and absence of the contralateral side lesion were positive factors for achieving bone union.

Previous multivariable studies on the conservative treatment of lumbar spondylolysis have reported the affected level of lesion and stage, other factors such as gap size of the lesion, lumbar lordosis, contralateral side stage, and reduced flexibility as unfavorable factors for bone union [12, 13].

However, the bone union rate of these studies ranged from 17 to 67%, which is lower than those reported by recent studies [3, 9, 12, 13, 15]. Furthermore, the previous study included main side terminal stage lesions, in which bone union is considered impossible to achieve. Therefore, the treatment results in the previous study are not in line with the current understanding of various factors affecting the conservative treatment of lumbar spondylolysis [12, 13].

Our study is novel compared to previous reports in that: 1. the “pre-lysis stage” lesions, which is a new stage concept reported recently and defined as a lesion showing no fracture line on CT but with a signal change on MRI [3, 14, 16, 18], were included, accounting for 31% of all lesions in this study and 2. patients with the main side terminal stage lesion, in which bone union is difficult or unable to be achieved based on previous studies [1, 3], are excluded.

To the best of our knowledge, this is probably the largest conservative treatment report evaluating bone union with CT, both in terms of the number of patients and lesions [1–3, 9–16]. Unlike reports that examine single factors, such as SBO, level of lesion, and stage, the present study has the advantage of uniformly evaluating all cases and collecting a sufficient quality of data and number of patients for multivariable analysis.

From the results of this study, the factors affecting bone union in patients with lumbar spondylolysis receiving conservative statement were the stage of the main and contralateral sides, which is consistent with the results of previous studies that reported the association of the fracture of bone ring structure in the vertebral arch in the axial plane with local instability and bone union rate in the conservative treatment of lumbar spondylolysis [9, 11].

In other words, the more advanced the stage of the lesion, the larger the fracture gap, the greater the instability, and the less favorable it is for bone union. Bilateral lesions with two fractures are less likely to heal than unilateral lesions with a single fracture of the bone ring. The rate of bone union is also greatly reduced when the gap between the two fractures is large due to the increased instability [9, 11].

The previously reported factors, such as the level of lesion, SBO, and age, were not significantly associated with bone union in the multivariable analysis.

L5 lesions are known to negatively affect bone union, and the association between bone union and level of the lesion cannot be completely ruled out based on the results of this study alone. It is considered analogous that the stage carries a lot of weight, and the level of lesions and SBO are only confounding factors [3, 9–13].

**Fig. 3:**
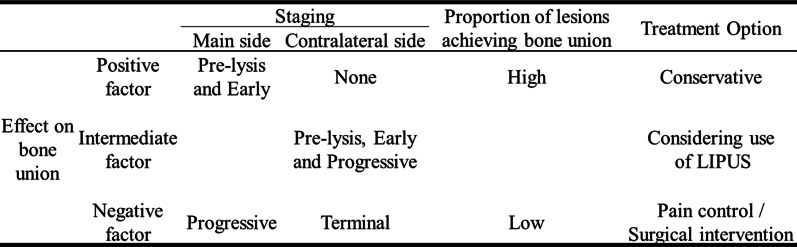
Treatment options according to factors affecting bone union. Evaluation of the main and contralateral staging at the initial visit will aid in selecting the treatment option. If there are more positive factors for bone union, conservative treatment should be selected. If there are more negative factors, pain control and surgical intervention should be considered. If intermediate, the use of low-intensity pulsed ultrasound (LIPUS) should be considered

Based on the results of the present study, the evaluation of factors affecting bone union at the time of the initial examination may assist in determining the treatment option (Fig. 3). As for imaging studies, only a single CT scan is sufficient to evaluate the stage of the lesion.

If there are more positive factors for bone union, the rate of bone union is expected to be higher; thus, conservative treatment aimed at achieving bone union is the first choice. If there are negative factors for bone union, low-intensity pulsed ultrasound, which has been reported to improve the rate of bone union, pain, and functional disability and shorten the treatment period, may be an option in addition to conventional conservative treatment [19–21].

In lesions that are expected to have a particularly low rate of bone union as they have many negative factors for bone union, if conservative treatment failed to achieve bone union, pain control with oral painkillers or interventional pain injections, early surgery due to pain, or repair surgery for lesions that became pseudoarthrosis may be effective depending on age and sports level [6–8, 22–25]. To further develop the treatment of lumbar spondylolysis, prospective studies on the ability to predict the rate of bone union at the time of the initial visit are a future prospect and challenge.

The present study had several limitations. All patients in the study received uniform imaging evaluations and the conservative treatment protocol. The number of dropouts was therefore relatively high (26%). The remaining patients were those with good treatment compliance, which could be a selection bias affecting the outcome of conservative treatment. In addition, all patients were treated in an outpatient setting, and not all patients were evaluated for height, weight, body mass index, or pain course using a uniform scale. The study assessed local factors such as the level and stage of the lesion, as well as demographic and clinical variables such as patient sex and age, and the presence of SBO. However, the sagittal alignment of the lumbar spine was not included to avoid too many variables in our multivariable analysis. Given the limited number of patients, this would have reduced the statistical power of our findings. Thus, alignment was omitted; but it should be noted that this was a major limitation of the study. Moreover, it was difficult to perform the imaging evaluation and conservative treatment in this study at all facilities due to cost, insurance, and other factors. Careful judgment should be exercised when using the results of this study in actual treatment. We have presented treatment options based on the present results; however, further research is needed to determine whether our approach can consistently improve bone union rates. A multicenter study is warranted to further include patients with varying background characteristics.

## Conclusions

In our multivariable analysis, the factors affecting bone union in patients receiving conservative treatment for lumbar spondylolysis were the main and contralateral side stages, with sex, age, level of lesion, and SBO having no significant effects. The main side progressive and contralateral side terminal stages were the negative factors for bone union, whereas the main side pre-lysis and early stages and the absence of contralateral side lesions were positive factors for bone union.

## Supplementary Information

**Additional file 1.** Patient characteristics and results of conservative treatment: age, sex, level of lesions, main and contralateral side stage, spina bifida occulta, treatment period, presence of bone union.

## Data Availability

The datasets generated during and analyzed during the current study are available from the corresponding author on reasonable request.

## Abbreviations

MRI: Magnetic resonance imaging
CT: Computed tomography
SBO: Spina bifida occulta
OR: Odds ratio
CI: Confidence intervals
LIPUS: Low-intensity pulsed ultrasound
